# Performance of Rapid Antigen Tests for COVID-19 Diagnosis: A Systematic Review and Meta-Analysis

**DOI:** 10.3390/diagnostics12010110

**Published:** 2022-01-04

**Authors:** Muhammad Fazli Khalid, Kasturi Selvam, Alfeq Jazree Nashru Jeffry, Mohamad Fazrul Salmi, Mohamad Ahmad Najib, Mohd Noor Norhayati, Ismail Aziah

**Affiliations:** 1 Institute for Research in Molecular Medicine (INFORMM), Health Campus, Universiti Sains Malaysia, Kubang Kerian 16150, Kelantan, Malaysia; fazlikhalid@usm.my (M.F.K.); kasturiselvam0612@gmail.com (K.S.); najib@student.usm.my (M.A.N.); 2 Faculty of Resource Science and Technology (FRST), Universiti Malaysia Sarawak, Kota Samarahan 94300, Sarawak, Malaysia; alfeqjazreenashru@yahoo.com (A.J.N.J.); mohdfazrul3277@gmail.com (M.F.S.); 3 Department of Family Medicine, School of Medical Sciences, Health Campus, Universiti Sains Malaysia, Kubang Kerian 16150, Kelantan, Malaysia; hayatikk@usm.my

**Keywords:** SARS-CoV-2, rapid antigen test, systematic review, sensitivity, specificity

## Abstract

The identification of viral RNA using reverse transcription quantitative polymerase chain reaction (RT-qPCR) is the gold standard for identifying an infection caused by SARS-CoV-2. The limitations of RT-qPCR such as requirement of expensive instruments, trained staff and laboratory facilities led to development of rapid antigen tests (RATs). The performance of RATs has been widely evaluated and found to be varied in different settings. The present systematic review aims to evaluate the pooled sensitivity and specificity of the commercially available RATs. This review was registered on PROSPERO (registration number: CRD42021278105). Literature search was performed through PubMed, Embase and Cochrane COVID-19 Study Register to search studies published up to 26 August 2021. The overall pooled sensitivity and specificity of RATs and subgroup analyses were calculated. Quality Assessment of Diagnostic Accuracy Studies 2 (QUADAS-2) was used to assess the risk of bias in each study. The overall pooled sensitivity and specificity of RATs were 70% (95% CI: 69–71) and 98% (95% CI: 98–98), respectively. In subgroup analyses, nasal swabs showed the highest sensitivity of 83% (95% CI: 80–86) followed by nasopharyngeal swabs 71% (95% CI: 70–72), throat swabs 69% (95% CI: 63–75) and saliva 68% (95% CI: 59–77). Samples from symptomatic patients showed a higher sensitivity of 82% (95% CI: 82–82) as compared to asymptomatic patients at 68% (95% CI: 65–71), while a cycle threshold (Ct) value ≤25 showed a higher sensitivity of 96% (95% CI: 95–97) as compared to higher Ct value. Although the sensitivity of RATs needs to be enhanced, it may still be a viable option in places where laboratory facilities are lacking for diagnostic purposes in the early phase of disease.

## 1. Introduction

Coronavirus disease 2019 (COVID-19), an infectious disease caused by severe acute respiratory syndrome coronavirus 2 (SARS-CoV-2), has evolved into a global pandemic and is still a major health concern around the world. The initial outbreak of SARS-CoV-2 started in Wuhan, China, in December 2019 [1,2]. As of 17 August 2021, SARS-CoV-2 infected over 211 million people and killed over 4.4 million people worldwide. Those infected with the SARS-CoV-2 experience a variety of symptoms, including fever, cough, exhaustion, shortness of breath, headache, sore throat, and loss of smell and taste [3,4,5]. The symptoms develop normally after 2 days to 2 weeks and last up to 3 weeks or longer for patients with mild to severe COVID-19 infection [1]. The SARS-CoV-2 infection can be divided into five stages. These stages are asymptomatic, mild, moderate, severe, and critical. Asymptomatic cases are infected individuals with no clinical symptoms. Cough, fever, nasal congestion, muscle soreness, and a sore throat are common symptoms in the early stages. Moderate stages showed mild or moderate clinical features, and the result of chest imaging revealed mild pneumonia. Shortness of breath and a drop in oxygen saturation below 93% are symptoms of severe phases, whereas respiratory failure and the requirement for mechanical ventilation, as well as other organ failure, necessitate ICU therapy.

The highly infectious SARS-CoV-2 is currently known to be transmitted to other people through respiratory droplets and aerosols exhibited by infected individuals [6]. COVID-19 sufferers’ exhalation, sneezing and coughing produce viral plumes containing thousands of droplets per cubic centimetre, causing transmission when an individual comes into contact with infected others [7]. Recently, the World Health Organization (WHO) officially declared COVID-19 as an air-borne transmission, which can be defined as droplets transmission with the presence of infectious agents in the droplet nuclei that may linger in the air for long periods of time [8]. Asymptomatic persons can also create and release vast quantities of droplets smaller than 1 μm during normal breathing and speech [9].

SARS-CoV-2 is severely affecting the global economics and mental health due to restrictions for preventing and controlling the disease transmission [10,11,12]. Rapid testing of those suspected of having COVID-19 infection is critical so that those who are infected can rapidly self-isolate, reducing the risk of SARS-CoV-2 spreading into the community. The reverse transcription quantitative polymerase chain reaction (RT-qPCR), which detects SARS-CoV-2 RNA in respiratory tract samples, is presently the gold standard for COVID-19 diagnosis [13,14]. Nonetheless, RT-qPCR requires competent workers, expensive equipment (thermal cycler with fluorimetry) and laboratory facilities (biosafety level 3, BSL-3) for RNA extraction. This could be a challenge in resource-constrained communities, such as those lacking basic laboratory facilities. Furthermore, RT-qPCR is time-consuming, with a sample-to-result turnaround time of up to 4 h, and it may not be acceptable to use in an emergency [15]. Rapid point-of-care tests (POCTs) that are less expensive and transportable in the field are needed to improve the surveillance of SARS-CoV-2 and control the outbreak.

The scientific community has achieved extraordinary progress in response to the limitations of RT-qPCR, resulting in the development of rapid diagnostic tools for improving SARS-CoV-2 surveillance. Rapid antigen tests (RATs) offer results more rapidly (approximately 15–30 min), are easy to perform and do not require highly trained staff and specialized laboratory equipment. Rapid SARS-CoV-2 antigen detection reduces the turnaround time for obtaining the results, allowing the immediate isolation of the infected individuals. As a result, the utilization of SARS-CoV-2 antigen detection will be beneficial for reducing the transmission rate.

In order to control infection sources, highly effective, rapid and low-cost diagnostic screening of a vulnerable population is required. This diagnostic capability also assists policymakers in assessing when and to what extent restrictions should be relaxed to help the economy recover. While numerous studies have presented antigen-based assays for SARS-CoV-2 surveillance, it is critical to analyse and make conclusions regarding the performance of these assays and the quality of these studies in a systematic manner. Previously, two systematic reviews have evaluated the pooled sensitivity and specificity of RATs for SARS-CoV-2 detection [16,17]. However, these reviews included only studies published up to 13 January and 30 April 2021, respectively. Since more diagnostic studies involving evaluation of the performance of RATs for SARS-CoV-2 have been published recently, it is important to update the available evidence so that more comprehensive findings can be obtained. These findings will help clinicians make better decisions about how to use antigen detection with the highest diagnostic specificity and sensitivity for contact monitoring. As a result, the aim of this systematic review was to update the diagnostic performance of rapid antigen tests for SARS-CoV-2.

## 2. Materials and Methods

This study followed the Preferred Reporting Items for Systematic Reviews and Meta-Analysis (PRISMA) guidelines [18]. The protocol of this systematic review and meta-analysis was registered in the PROSPERO database (CRD42021278105).

### 2.1. Eligibility Criteria

This systematic review and meta-analysis included (1) only peer-reviewed original articles on antigen-based detection for SARS-CoV-2 or COVID-19; (2) the reference standard for detection assays must be RT-qPCR; (3) full text is accessible (in English only); and (4) offer adequate data to calculate the number of false positives, true positives, true negatives and false negatives on antigen detection in comparison to a standard reference test. Antibody tests, nucleic acid tests, review papers, and studies that did not involve clinical samples were all excluded from the study.

### 2.2. Search Strategy

The peer reviewed articles were searched on 26 August 2021 without restricting the publication date. The articles were searched through PubMed, Embase and Cochrane COVID-19 Study Register by using several keywords with the following search string: [COVID-19 OR SARS-CoV-2] AND [antigen] AND [specificity OR sensitivity]. The titles, abstracts and full text of relevant studies were screened manually by five researchers (M.F.K., A.J.N.J., M.F.S., M.A.N. and K.S.).

### 2.3. Data Analysis

Three authors (M.F.K., A.J.N.J. and M.F.S.) individually extracted the number of true positives (TP), true negatives (TN), false positives (FP) and false negatives (FN) from each study and entered them into an Excel datasheet. Disagreeable findings were discussed, and when in doubt, the authors sought verification. The sensitivity and specificity for each of the studied antigen were calculated using RT-qPCR as the reference standard. The number of true positive outcomes was divided by the sum of true positive and false negative outcomes to calculate the sensitivity (1). The number of true negative outcomes was divided by the sum of true negative and false positive outcomes to calculate the specificity (2). Forest plots were used to demonstrate the comparative performance of the rapid antigen tests:

(1) $$Sensitivity=\frac{\mathrm{TP}}{\mathrm{TP}+\mathrm{FN}}$$

(2) $$Specificity=\frac{\mathrm{TN}}{\mathrm{TN}+\mathrm{FP}}$$

### 2.4. Quality Assessment

The quality of each study was assessed using Quality Assessment of Diagnostic Accuracy Studies 2 (QUADAS-2) tool [19]. Patient selection, index test, reference standard and flow and timing are the four core domains of the QUADAS-2 tool Table 1). The risk of bias was classified as low, high or unclear for each domain. Five authors (M.F.K., A.J.N.J., M.F.S., M.A.N. and K.S.) independently performed the assessment to judge the quality of each study. Disagreements among the authors were resolved by discussion.

## 3. Results

### 3.1. Search Results

**Figure 1:**
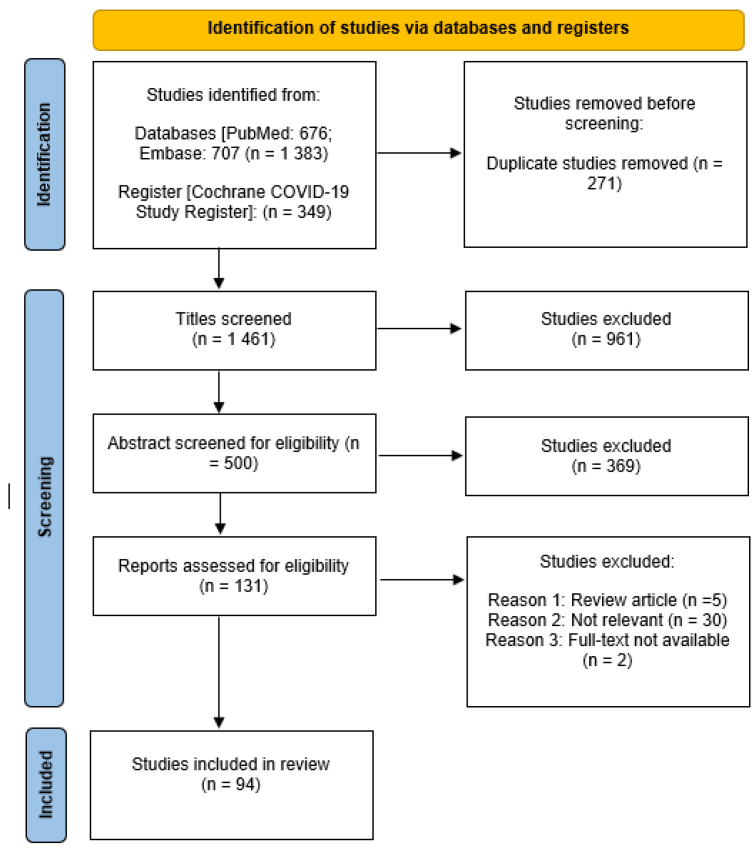
The preferred reporting items for a systematic review and meta-analysis (PRISMA) flow diagram.

A total of 1732 articles were identified through databases and register searching (Figure 1). Of these, 271 duplicates were identified and removed using data tool in Excel. After screening the titles, 961 articles that were not primary research articles and unrelated to antigen-based detection for COVID-19 were excluded. Of the 500 abstracts screened, 369 articles that did not meet the basis of selection criteria were excluded. After screening the full text articles, 37 articles were excluded. The remaining 94 studies that fulfilled our selection criteria were included in this systematic review.

### 3.2. Characteristics of the Included Studies

The characteristics of the 94 studies reporting the performance of rapid antigen tests (RATs) for SARS-CoV-2 were summarized in Table 2. Most of the studies (n = 84) were published in 2021, and the remaining studies were published in 2020. The majority of the studies employed clinical samples from European countries which include Germany (n = 14), Spain (n = 11), Italy (n = 10), the Netherlands (n = 7), France (n = 6), Belgium (n = 5) and Switzerland (n = 3). In Asia, most studies were conducted in Japan (n = 7), followed by China (n = 3) and India (n = 2). The United States (n = 8) and Chile (n = 3) were among the American countries reported the performance of RAT for SARS-CoV-2. A total of 25 studies used Panbio™ COVID-19 Ag RDT (Abbott, Jena, Germany), 11 used STANDARD Q COVID-19 Ag Home Test (SD Biosensor, Seoul, South Korea), 10 used SARS-CoV-2 Rapid Ag Test (Roche, Basel, Switzerland) and 9 used Lumipulse G SARS-CoV-2 Ag (Fujirebio, Tokyo, Japan). Most of the studies (n = 71) used nasopharyngeal swabs, 12 used pools of nasopharyngeal and throat swabs, 4 used nasal swabs, 3 used saliva, 2 used throat swabs, 1 study used a pool of nasopharyngeal and nasal swabs and 1 study compared the performance of nasopharyngeal swabs, saliva and sputum.

### 3.3. Quality of Articles

**Figure 2:**
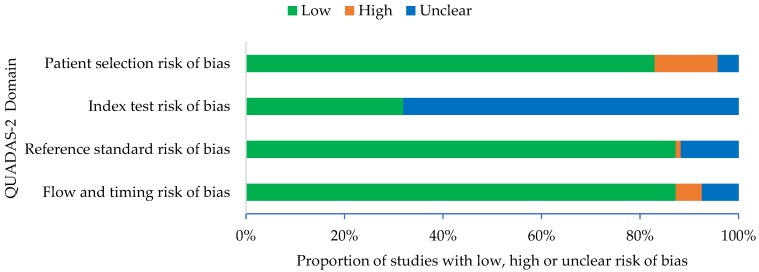
QUADAS-2 criteria for 94 studies included in this systematic review.

The QUADAS checklist was completed for all included studies (Supplementary Material Table S1). The QUADAS-2 criteria for 94 studies included in this systematic review were presented in Figure 2. Majority of the included studies (83%) have low risk of patient selection bias. Twelve (13%) studies have high risk of patient selection bias due to the case–control study design. These studies specifically recruited clinical samples known to be uninfected or infected with coronavirus. The remaining studies (4%) have unclear risk of patient selection bias. These studies were not case–control but provided insufficient details about the inclusion and exclusion criteria. About 68% (64 out of 94 studies) have unclear risk of index bias due to unclear information on whether the index test results were interpreted with knowledge of reference test results. The remaining studies (32%) have low risk of index bias as the reference tests ware blinded from each other and results were recorded independently by two readers.

With regards to the reference standard risk of bias, almost all the studies (87%) have low risk of bias, as these studies used similar RT-PCR as the reference standard and the reference tests results were interpreted without knowledge of index test results. Eleven studies (12%) have unclear risk of reference standard bias due to these studies did not provide enough information about whether reference standard results were interpreted without knowledge of the results of the index test. Only 1% of the studies has high risk of reference standard bias because the reference standard results were interpreted with knowledge of the results of the index test. Most of studies (87%) have low risk of flow and timing bias. Seven studies (8%) have unclear risk of flow and timing bias due to no information on whether the samples for a reference test and the index test were taken at the same time. Only five studies (5%) have high risk of flow and timing bias. One of the five studies has high risk of flow and timing bias because samples were collected from the same patients at multiple time points. Another three studies have high risk of flow and timing bias due to the use of different samples for index and reference test and one more due to use of different standard references.

### 3.4. Meta-Analysis of the Sensitivity and Specificity of Rapid Antigen Tests

**Figure 3:**
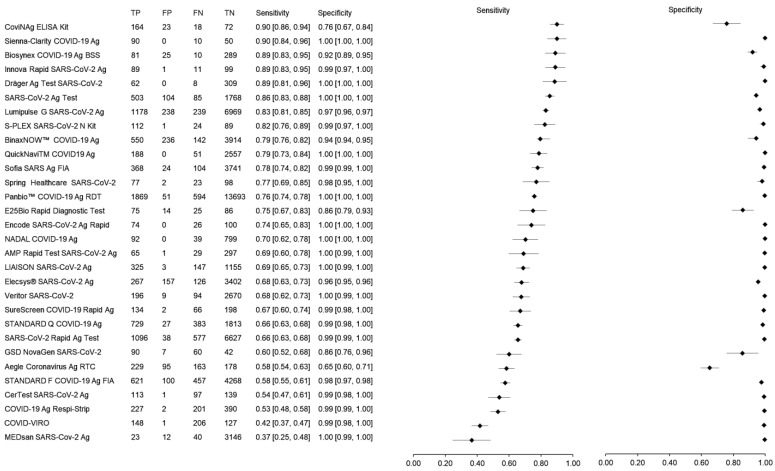
The forest plot of sensitivity and specificity of rapid antigen tests for SARS-CoV-2.

A total of 74,445 samples tested using 30 different rapid antigen tests (RATs) and confirmed with RT-PCR as reference test were included in the meta-analysis. The sensitivity and specificity of the RATs ranged from 37% to 90% and 65% to 100%, respectively (Figure 3). Of the 30 RATs analyzed, 52% (n = 16) reported at least 70% sensitivity. CoviNAg ELISA Kit (XEMA, Moscow, Russia) and Sienna-Clarity COVID-19 Ag RTC (Salofa Oy, Salo, Finland) showed highest sensitivity of 90% while MEDsan SARS-Cov-2 Ag (MEDsan GmbH, Hamburg, Germany) showed lowest sensitivity of 37%. Nonetheless, when those kits tested using small number of samples were excluded from analysis, SARS-CoV-2 Ag Test (LumiraDx GmbH, Cologne, Germany) showed the highest sensitivity of 86% followed by Lumipulse G SARS-CoV-2 Ag (Fujirebio, Tokyo, Japan) and BinaxNOW™ COVID-19 Ag Self-Test (Abbott, Jena, Germany) with sensitivity of 83% and 79%, respectively.

Meanwhile, for specificity, almost all RATs reported over 90% specificity with only four (13%) showed specificity below 90%. These include CoviNAg ELISA Kit (XEMA, Moscow, Russia), E25Bio Rapid Diagnostic Test (E25Bio, Cambridge, USA), GSD NovaGen SARS-CoV-2 (Eurofins Technologies, Budapest, Hungary) and Aegle Coronavirus Ag RTC (Bio-Rad, California, United States). Panbio™ COVID-19 Ag RDT (Abbott, Jena, Germany) was observed to be the most studied kit for SARS-CoV-2 detection with 16,207 samples were included from 25 different studies. The pooled sensitivity and specificity of Panbio™ COVID-19 Ag RDT was 75.9% and 99.6%.

### 3.5. Performance of the Rapid Antigen Tests Based on Subgroup Analyses

The overall pooled sensitivity and specificity of RATs were 70% (95% CI: 69–71) and 98% (95% CI: 98–98), respectively (Table 3). For subgroup analysis of the RATs based on specimens, a total of 65 studies were included after excluding studies utilizing combination of nasopharyngeal and throat swabs. Nasal swabs showed the highest sensitivity of 83% (95% CI: 80–86), whereas nasopharyngeal swabs showed a sensitivity of 71% (95% CI: 70–72), throat swabs 69% (95% CI: 63–75) and saliva 68% (95% CI: 59–77). Comparably high specificities were observed among all four specimens, ranging from 97% to 99%. 

Subgroup analysis based on the presence of symptoms showed RATs gives higher sensitivity (82%) among symptomatic as compared with asymptomatic (68%). The specificity of RATs was similar in both symptomatic and asymptomatic patients (98%). Cycle threshold (Ct) values are inversely correlated with the viral load in a specimen. Among those patients with higher viral load (Ct value ≤25), the RATs showed sensitivity and specificity of 96% and 99%, respectively. Meanwhile, the sensitivity of RATs dropped to 69% when used on patients with low viral load (Ct value >25). A subgroup analysis of the RATs based on countries showed sensitivity and specificity ranging from 58% to 87% and 96% to 100%, respectively. The sensitivity was observed to be highest in China (87%) followed by Italy (81%), Chile (77%), the United States (77%), Belgium (73%), Japan and the Netherlands (72%) and Spain (71%). On the other hand, the sensitivity was observed to be lower than average (70%) in Germany, France and India with all showed sensitivity of 58%. In regard to specificity, comparably high specificities, ranging from 96% to 100% were observed in all countries, except in Belgium (84%).

After excluding the case–control studies, the overall pooled sensitivity and specificity of RATs were 72% (95% CI: 71–73) and 98% (95% CI: 98–98), respectively (Table 4). Meanwhile, the diagnostic performance of different specimens showed similar result to the subgroups analysis without excluding case–control studies. For analysis based on symptoms, the RATs performance after excluding case–control studies showed lower sensitivity of 80% (95% CI: 78–81) and higher specificity of 99% (95% CI: 99–99). Similarly, the RATs performance showed lower sensitivity and higher specificity when tested for samples from those patients with Ct value >25. The performance of RATs for asymptomatic and patients with Ct value ≤25 remain similar.

Further analysis, which includes comparative performance of studies that blinded the index test and studies that did not blind the index test were performed (Table 5). Studies where the index text was blinded to the results of the reference standard had a sensitivity and specificity of 72% and 97%, respectively. Meanwhile, studies where the index test was not blinded to the results of the reference standard has a sensitivity and specificity of 72% and 97%, respectively.

## 4. Discussion

Recently, there have been two systematic reviews on the performance of RATs for SARS-CoV-2 detection [16,17]. These reviews only included studies up to 13 January and 30 April 2021. Our review provided more comprehensive analysis on the performance of RATs until 26 August, following the inclusion of 94 studies. Nonetheless, most of the studies still from European countries such as Germany, Spain, Italy, the Netherlands, France, Belgium and Switzerland. Lack of studies from West and Southeast Asian countries, South America and Africa, highlighting the current gap pertaining to understanding the RATs performance in such geographical areas. A similar observation was reported by the previous study, in which, most of the included studies were from Germany, Spain and Italy [17]. The plausible reason may be attributed to the fact that most kits are manufactured in European countries; thus, such test kits were easier to obtain in those countries as compared to others where supply shortages are commonly reported. The second reason is that these countries were badly affected by COVID-19 in the beginning of the outbreak. Therefore, the COVID-19 RAT is becoming popular across European countries as governments’ efforts to slow the spreading of the virus by tracking infected individuals.

Panbio™ COVID-19 Ag RDT (Abbott, Jena, Germany) and STANDARD Q COVID-19 Ag Home Test (SD Biosensor, Seoul, South Korea) were used in the majority of studies (25 and 11 studies, respectively) followed by SARS-CoV-2 Rapid Ag Test (Roche, Basel, Switzerland) and Lumipulse G SARS-CoV-2 Ag (Fujirebio, Tokyo, Japan). This could be due to the fact that Panbio™ COVID-19 Ag RDT (Abbott, Jena, Germany) and STANDARD Q COVID-19 Ag Home Test (SD Biosensor, Seoul, South Korea) are two RAD kits that are currently included under the ‘WHO Emergency Use Listing for In vitro diagnostics (IVDs) Detecting SARS-CoV-2′ [113]. There is still a lack of evaluation for newly developed test kits such as CoviNAg ELISA Kit (XEMA, Moscow, Russia) and Sienna-Clarity COVID-19 Ag RTC (Salofa Oy, Salo, Finland). Therefore, future diagnostic evaluation studies should include the newly developed test kits so that more data can be obtained for future comparative analyses. 

Immunochromatography, which involves spotting antibodies onto nitrocellulose membranes that interact with specific antigens in patient samples is the basis of RATs. The antigen–antibody interaction can be visualised manually or by using an immunofluorescence machine reader. The genome of SARS-CoV-2 comprises genes the responsible for four structural proteins such the spike (S), envelope (E), membrane (M) and nucleocapsid (N) [114]. N protein is frequently employed as a target analyte in RATs for COVID-19 diagnosis, as shown in Table 2. N-protein is mostly expressed during the early stages of SARS-CoV-2 infection and has the least amount of variation in its gene sequence, indicating that it is a stable protein [115]. 

Most studies used nasopharyngeal swabs as specimens examined for evaluating the performance of the test kits. Only a few studies used nasal swabs, saliva and throat swabs. This observation signaling the need for more studies using such specimens so that alternative sampling approaches for the rapid detection of SARS-CoV-2 could be identified. Subgroup analysis of the test kits based on specimens showed nasal swabs gave the highest sensitivity of 83% (95% CI: 80–86) followed by nasopharyngeal swabs 71% (95% CI: 70–72), throat swabs 69% (95% CI: 63–75) and saliva 68% (95% CI: 59–77). Interestingly, nasopharyngeal swabs are currently considered as the gold standard specimen for SARS-CoV-2 laboratory diagnosis. This finding may suggest that nasal swab could replace nasopharyngeal swabs as the gold standard specimen. However, it is important to note that the average sensitivity of the nasal swabs was calculated only based on 2148 samples as compared to 38548 samples for nasopharyngeal swabs. Thus, evaluation using more samples are still needed to provide a comprehensive conclusion regarding the nasal swabs’ performance. Lower sensitivity for throat swabs was in agreement with a recent study on the performances of the different sampling approaches for SARS-CoV-2, which reported lower sensitivity by throat swabs (68%) as compared to nasal swabs and saliva [116].

The meta-analysis shows that the sensitivity and specificity of the 30 RATs ranged from 37% to 90% and 65% to 100%, respectively, whereas the overall pooled sensitivity and specificity of RATs were 70% (95% CI: 69–71) and 98% (95% CI: 98–98), respectively. Based on sample size, the SARS-CoV-2 Ag Test (LumiraDx GmbH, Cologne, Germany) showed the highest sensitivity of 86% followed by the Lumipulse G SARS-CoV-2 Ag (Fujirebio, Tokyo, Japan) and BinaxNOW™ COVID-19 Ag Self-Test (Abbott, Jena, Germany) with sensitivities of 83% and 79%, respectively. Furthermore, most of the studies showed a high level of specificity (greater than 90%). The WHO recommended that the minimum performance requirements of RATs must be ≥ 80% sensitivity and ≥97% specificity. The studies included in this review have showed a wide range of sensitivity and specificity. The different results could be related to various study methods, RAT kits manufacturers, patient selection, types of specimens and the stage of disease at the time of sample collection. Based on the meta-analysis, the RAT kits that meet WHO criteria are Sienna-Clarity COVID-19 Ag RTC (Salofa Oy, Salo, Finland), Biosynex COVID-19 Ag BSS Test (Biosynex, Strasbourg, France), Innova Rapid SARS-CoV-2 Ag Test (Xiamen Biotime Biotechnology, Fujian, China), Dräger Ag Test SARS-CoV-2 (Dräger, Lübeck, Germany), SARS-CoV-2 Ag Test (LumiraDx GmbH, Cologne, Germany), Lumipulse G SARS-CoV-2 Ag (Fujirebio, Tokyo, Japan), S-PLEX SARS-CoV-2 N Kit (MesoScale Diagnostics, Maryland, USA) and BinaxNOW™ COVID-19 Ag Self-Test (Abbott, Jena, Germany). 

Subgroup analysis of the RATs based on countries showed sensitivity and specificity ranging from 58% to 87% and 96% to 100%, respectively. The sensitivity was observed to be highest in China (87%) followed by Italy (81%), Chile (77%), the United States (77%), Belgium (73%), Japan and the Netherlands (72%) and Spain (71%). On the other hand, the sensitivity was observed to be lower than average (70%) in Germany, France and India with all showing a sensitivity of 58%. The previous systematic review reported that the sensitivity of RATs in the population of Europe and America was higher as compared to that of Asia and Africa [17]. Their finding was different with our finding. One plausible reason is that the RATs evaluated previously were manufactured from Europe and America which may affect the test performance in Asia and Africa after repeated freeze–thaw procedures during transportation [117]. In regard to specificity, comparably high specificities, ranging from 96% to 100%, were observed in all countries, except in Belgium (84%). 

According to the WHO, RATs should be prioritized for use in symptomatic individuals who meet the COVID-19 case definition, as well as to test asymptomatic individuals at high risk of infection, particularly in settings where NAAT testing capacity is limited. Thus, this review also analysed the sensitivity and specificity of RATs based on symptoms. RATs showed similar specificity (98%) for symptomatic and asymptomatic patients. However, the sensitivity of RATs for symptomatic patients was greater than asymptomatic patients (82% vs. 68%). A similar finding was reported by the previous systematic reviews, in which, the sensitivity of RATs is higher when used for symptomatic patients [16,17]. In addition, there is a clear association between Ct values of RT-qPCR and RATs’ sensitivity and specificity. The lower the Ct value (≤25), the greater the sensitivity and specificity of RATs, whereas the higher the Ct value (<25), the lower the sensitivity and specificity of RATs. Ct values, on the other hand, cannot be directly compared between tests and must be interpreted with caution because they are impacted by sample type, sample collection timing, and assay design [118].

The severity of the disease, the timing of sample collection, the types of samples, and sample handling techniques all influence antigen levels in samples [20]. It is hard to determine if the difference in observed sensitivity is due to the test’s performance or the qualities of the samples utilized in the test without this information. Unfortunately, the majority of the studies included in this review did not provide information on antigen levels in the samples. Information about disease severity and sample collection timing are often missing. Future research should include this information to enable for a more accurate assessment of diagnosis test performance as well as the identification of their actual limitations. Throughout this systematic review, our study identified the relevant peer-review articles published to reach the objective findings about the performances of the antigen detection for diagnostic of COVID-19. This study followed the PRISMA guidelines to reduce the risk of bias and meet the objective of the review. The use of several pre-determined keywords and guidelines in this study during the screening process can assure the reproducibility of this systematic review. The screening process that started from the title screening followed by abstract and full text screening was documented properly to avoid any risk of bias in the systematic review. 

Based on the quality assessment of the QUADAS-2, most of the included studies showed a low of bias. However, there were several studies that indicate high risk of bias for the QUADAS-2 assessment. The high risk in the patient selection was due to the case–control study design, while the unclear risk of the patient selection was due to insufficient details about the patient inclusion and exclusion criteria. For the risk of the index test, most of the included studies were unclear as the authors did not mention whether the index test results were interpreted with knowledge of the reference test results. The high risk of the reference standard bias was due to the results that were not interpreted with knowledge of the result of the index test. For the risk of the flow and timing bias, high risk of the bias due to the several reference standards or collected samples for the index and references tests at separate time. 

Nevertheless, our systematic review has three main limitations. First, this systematic review revealed that there was considerable publication bias in the included studies as the study on the performances of the antigen detection of the SARS-CoV-2 were still in the early stages of development and almost all reported diagnostics performance assessments were done and published by the same research group. We anticipated that such bias will be reduced after these antigen performances were completely evaluated by various and independent research teams. Second, the presence of the SARS-CoV-2 antigen in the patients does not always indicate the presence of the viable virus, and we need to examine whether SARS-CoV-2 antigen-positive patients are contagious to other people. Lastly, the possibility of protein mutations in SARS-CoV-2 variants, including the newly revealed Omicron, affecting the sensitivity and specificity of RATs is not reported in this review.

## 5. Conclusions

In conclusion, our systematic review and meta-analysis revealed the current performance of RATs for SARS-CoV-2 detection. Overall diagnostic sensitivity and specificity of these RATs were 70% and 98%, respectively. Quality assessment showed majority of the studies have low risk of bias. However, several studies showed high risk of patient selection bias due to case-control study design and high risk of flow and timing bias due to the use of different samples for index and reference test and use of different standard references. Regarding index test risk of bias, the majority did not mention whether or not the authors performed double-blinded index test. Future study should attempt to perform double-blinded index test as such improvement in study design would reduce index test risk of bias. CoviNAg ELISA Kit (XEMA, Moscow, Russia) and Sienna-Clarity COVID-19 Ag RTC (Salofa Oy, Salo, Finland) had the highest diagnostic sensitivity among all RATs, while MEDsan SARS-Cov-2 Ag (MEDsan GmbH, Hamburg, Germany) had the lowest diagnostic sensitivity. More studies from the Middle East and Southeast Asian, South American, and African countries are warranted so that a comprehensive subgroup analysis based on regions can be performed. Nasal swabs showed the highest sensitivity followed by nasopharyngeal swabs, throat swabs and saliva. The RATs showed higher sensitivity for those patients with symptoms and Ct value ≤25. Comparative performance of the RATs using less invasive sampling approaches is still lacking. Improvement in these key areas would help to boost acceptability and accessibility for large practical of RATs for rapid surveillance of SARS-CoV-2, allowing immediate isolation of the infected individuals and reducing the disease’s transmission.

## Figures and Tables

**Table 1:** QUADAS-2 risk of bias assessment criteria.

Domains	Criteria for Low Risk Assessment
Patient selection	Patient enrolment strategy is specified and free of bias. A case–control design and inappropriate exclusions are avoided.
Index test	The index test results are interpreted without knowledge of the results of the reference standard. The conduct or interpretation of the index test does not introduce bias.
Reference standard	The reference standard correctly classifies the target condition. The reference standard results are interpreted without knowledge of the results of the index test. The reference standard, its conduct or its interpretation do not introduce bias.
Flow and timing	There is an appropriate interval between the index test(s) and reference standard. All patients receive the same reference standard. All patients included in the analysis and patient flow do not introduce bias.

**Table 2:** Characteristics of included studies.

	Tests Investigate	Target	Sample	Sample Size	Sensitivity	Specificity	Location	References
1	STANDARD Q COVID-19 Ag Home Test (SD Biosensor, Seoul, South Korea)	N	NPS and TS	454	98.33%	98.73%	Thailand	[20]
2	Biocredit Covid-19 Ag Detection Kit (RapiGEN, Gyeonggi-do, Korea)	N	NPS and TS	35	34.30%	NR	Hong Kong	[21]
			SP	45	11.10%	NR		
			S	45	40.00%	NR		
3	COVID-19 Ag Respi-Strip (Coris BioConcept, Gembloux, Belgium)	N	NPS	148	30.20%	100%	Belgium	[22]
4	Espline SARS-CoV-2 (Fujirebio, Tokyo, Japan)	N	S	103	11.70%	NR	Japan	[23]
5	Sofia SARS Ag FIA (Quidel, San Diego, USA)	N	NPS and TS	127	93.90%	100%	Chile	[24]
6	SARS-CoV-2 Rapid Ag Test (Roche, Basel, Switzerland)	N	NPS	150	70.70%	96.00%	Germany	[25]
7	Panbio™ COVID-19 Ag RDT (Abbott, Jena, Germany)	N	NPS	412	79.60%	100%	Spain	[26]
8	Panbio™ COVID-19 Ag RDT (Abbott, Jena, Germany)	N	NPS	634	48.10%	100%	Spain	[27]
9	Sofia SARS Ag FIA (Quidel, San Diego, USA)	N	NPS (asymptomatic)	871	41.20%	98.40%	United States	[28]
			NPS (symptomatic)	227	80.00%	98.90%		
10	In house	N	NPS	251	75.60%	100%	China	[29]
11	COVID-19 Ag Respi-Strip (Coris BioConcept, Gembloux, Belgium)	N	NPS	138	50.00%	100%	France	[30]
12	Panbio™ COVID-19 Ag RDT (Abbott, Jena, Germany)	N	NPS	255	73.30%	NR	Spain	[31]
13	STANDARD F COVID-19 Ag FIA (SD Biosensor, Seoul, South Korea)	N	NPS and TS	741	45.40%	97.80%	Germany	[32]
	SARS-CoV-2 Rapid Ag Test (Roche, Basel, Switzerland)	N	NPS and TS	831	50.30%	97.70%		
14	Lumipulse G SARS-CoV-2 Ag (Fujirebio, Tokyo, Japan)	N	NPS	548	91.70%	98.50%	Japan	[33]
15	STANDARD Q COVID-19 Ag Home Test (SD Biosensor, Seoul, South Korea)	N	NPS	262	70.00%	92.00%	Uganda	[34]
16	S-PLEX SARS-CoV-2 N Kit (MesoScale Diagnostics, Maryland, USA)	N	NPS	226	82.00%	99.00%	United States	[35]
17	Panbio™ COVID-19 Ag RDT (Abbott, Jena, Germany)	N	NPS	1620	45.40%	99.80%	Spain	[36]
18	Lumipulse G SARS-CoV-2 Ag (Fujirebio, Tokyo, Japan)	N	NPS	274	75.70%	96.00%	Japan	[37]
19	Aegle Coronavirus Ag RTC (Bio-Rad, California, United States)	N	NPS	199	59.30%	100%	Belgium	[38]
	GSD NovaGen SARS-CoV-2 (Eurofins Technologies, Budapest, Hungary)	N	NPS	199	60.00%	85.70%		
	Aegle Coronavirus Ag RTC (Bio-Rad, California, United States)	N	NPS	199	61.10%	100%		
20	STANDARD Q COVID-19 Ag Home Test (SD Biosensor, Seoul, South Korea)	N	NPS	1223	37.84%	100%	Slovakia	[39]
21	STANDARD Q COVID-19 Ag Home Test (SD Biosensor, Seoul, South Korea)	N	NPS and TS	2032	42.86%	99.89%	Germany	[40]
22	CerTest SARS-CoV-2 Ag (Certest Biotec, Zaragoza, Spain)	N	NPS	320	53.50%	100%	Spain	[41]
	Panbio™ COVID-19 Ag RDT (Abbott, Jena, Germany)	N	NPS	320	60.00%	100%		
23	Lumipulse G SARS-CoV-2 Ag (Fujirebio, Tokyo, Japan)	N	NPS and TS	4266	86.60%	97.30%	Italy	[42]
24	CoviNAg ELISA Kit (XEMA, Moscow, Russia)	N	NPS	277	90.10%	75.80%	Russia	[43]
25	Lumipulse G SARS-CoV-2 Ag (Fujirebio, Tokyo, Japan)	N	NPS	226	92.60%	90.80%	Italy	[44]
	Lumipulse G SARS-CoV-2 Ag (Fujirebio, Tokyo, Japan)	N	NPS	1738	100%	94.80%		
26	Innova Rapid SARS-CoV-2 Ag Test (Xiamen Biotime Biotechnology, Fujian, China)	N	NPS and TS	200	89.00%	99.00%	United Kingdom	[45]
	Spring Healthcare SARS-CoV-2 Ag RTC (Shanghai ZJ Bio-Tech, Shanghai, China)	N	NPS	200	77.00%	98.00%		
	E25Bio Rapid Diagnostic Test (E25Bio, Cambridge, USA)	N	NPS	200	75.00%	86.00%		
	Encode SARS-CoV-2 Ag Rapid Test Encode SARS-CoV-2 Ag Rapid Test Device (Zhuhai Encode Medical Engineering, Zhuhai, China)	N	NS and TS	200	74.00%	100%		
	SureScreen COVID-19 Rapid Ag Test (SureScreen Diagnostics, Derby, UK)	N	NPS	200	69.00%	98.00%		
27	Lumipulse G SARS-CoV-2 Ag (Fujirebio, Tokyo, Japan)	N	NPS	529	84.80%	97.90%	Japan	[46]
	SARS-CoV-2 Rapid Ag Test (Roche, Basel, Switzerland)	N	NPS	637	70.00%	100%		
28	Lumipulse G SARS-CoV-2 Ag (Fujirebio, Tokyo, Japan)	N	S	305	77.80%	99.60%	Japan	[47]
29	Panbio™ COVID-19 Ag RDT (Abbott, Jena, Germany)	N	NPS	402	81.00%	99.10%	Switzerland	[48]
30	LIAISON SARS-CoV-2 Ag (DiaSorin, Saluggia, Italy)	N	NPS	196	40.20%	100%	Germany	[49]
31	Biosynex COVID-19 Ag BSS Test (Biosynex, Strasbourg, France)	N	NPS	97	89.70%	46.20%	Belgium	[50]
	SARS-CoV-2 Ag Card (Biotical Health, Madrid, Spain)	N	NPS	98	67.20%	100%		
	Aegle Coronavirus Ag RTC (Bio-Rad, California, United States)	N	NPS	98	82.80%	92.50%		
	Panbio™ COVID-19 Ag RDT (Abbott, Jena, Germany)	N	NPS	97	78.90%	100%		
	STANDARD F COVID-19 Ag FIA (SD Biosensor, Seoul, South Korea)	N	NPS	98	82.80%	100%		
32	QuickNaviTM COVID19 Ag (Otsuka, Japan)	N	NS	862	72.50%	100%	Japan	[51]
33	COVID-19 Ag Detection Kit (Colloidal Gold-CG)	N	NPS	358	89.47%	99.59%	Slovenia	[52]
34	LIAISON SARS-CoV-2 Ag (DiaSorin, Saluggia, Italy)	N	NPS	182	70.00%	100%	Germany	[53]
35	AMP Rapid Test SARS-CoV-2 Ag (AMP Diagnostics, Graz, Austria)	N	NPS	392	69.15%	99.66%	Austria	[54]
36	Panbio™ COVID-19 Ag RDT (Abbott, Jena, Germany)	N	NPS	1108	86.80%	99.90%	Germany	[55]
37	Sienna-Clarity COVID-19 Ag RTC (Salofa Oy, Salo, Finland)	N	NPS	150	90.00%	100%	France	[56]
38	COVID-19 Ag Respi-Strip (Coris BioConcept, Gembloux, Belgium)	N	NPS	50	30.70%	100%	Italy	[57]
39	Panbio™ COVID-19 Ag RDT (Abbott, Jena, Germany)	N	NPS	433	86.70%	100%	The Netherlands	[58]
40	SARS-CoV-2 Rapid Ag Test (Roche, Basel, Switzerland)	N	NPS	321	73.15%	100%	Italy	[59]
41	SARS-CoV-2 Ag Test (LumiraDx GmbH, Cologne, Germany)	N	NPS and NS	907	90.30%	92.10%	Italy	[60]
42	STANDARD F COVID-19 Ag FIA (SD Biosensor, Seoul, South Korea)	N	NPS	842	69.86%	99.61%	Chile	[61]
43	STANDARD Q COVID-19 Ag Home Test (SD Biosensor, Seoul, South Korea)	N	NPS	369	75.90%	100%	Mexico	[62]
44	Panbio™ COVID-19 Ag RDT (Abbott, Jena, Germany)	N	TS	402	81.00%	99.00%	United States	[48]
45	STANDARD Q COVID-19 Ag Home Test (SD Biosensor, Seoul, South Korea)	N	NPS	120	58.10%	100%	Serbia	[63]
46	COVID-19 Ag Respi-Strip (Coris BioConcept, Gembloux, Belgium)	N	NPS	484	71.96%	99.32%	India	[64]
47	COVID-VIRO (AAZ-LMB Boulogne-Billancourt, France)	N	NPS	248	96.70%	100%	France	[65]
48	Panbio™ COVID-19 Ag RDT (Abbott, Jena, Germany)	N	NPS	1362	71.40%	99.80%	Spain	[66]
49	Panbio™ COVID-19 Ag RDT (Abbott, Jena, Germany)	N	NPS	356	60.00%	100%	Spain	[67]
	STANDARD F COVID-19 Ag FIA (SD Biosensor, Seoul, South Korea)	N	NPS	356	66.50%	97.30%		
50	Veritor SARS-CoV-2 (BectonDickinson, New Jersey, USA)	N	TS	351	94.12%	100%	The Netherlands	[68]
51	Lumipulse G SARS-CoV-2 Ag (Fujirebio, Tokyo, Japan)	N	S	127	52.40%	94.10%	Italy	[69]
52	Panbio™ COVID-19 Ag RDT (Abbott, Jena, Germany)	N	NPS	535	85.50%	100%	United States	[70]
	STANDARD Q COVID-19 Ag Home Test (SD Biosensor, Seoul, South Korea)	N	NPS	529	89.00%	100%		
53	Dräger Ag Test SARS-CoV-2 (Dräger, Lübeck, Germany)	N	NS	379	88.60%	99.70%	Germany	[71]
54	MEDsan SARS-Cov-2 Ag (MEDsan GmbH, Hamburg, Germany)	N	NPS	806	36.51%	99.61%	Germany	[72]
	Panbio™ COVID-19 Ag RDT (Abbott, Jena, Germany)	N	NPS	1029	46.67%	99.62%		
	NADAL COVID-19 Ag (Nal Von Minden GmbH, Moers, Germany)	N	NPS	3221	56.62%	100%		
55	Sofia SARS Ag FIA (Quidel, San Diego, USA)	N	NPS	188	80.00%	100%	Finland	[73]
	STANDARD Q COVID-19 Ag Home Test (SD Biosensor, Seoul, South Korea)	N	NPS	198	81.00%	100%		
	Panbio™ COVID-19 Ag RDT (Abbott, Jena, Germany)	N	NPS	190	83.00%	100%		
56	CerTest SARS-CoV-2 Ag (Certest Biotec, Zaragoza, Spain)	N	NPS	80	55.00%	97.50%	The Netherlands	[74]
	SARS-CoV-2 Rapid Ag Test (Roche, Basel, Switzerland)	N	NPS	80	62.50%	87.50%		
	Romed SARS-CoV-2 Ag RTC (Oostveen Medical BV Van, Wilnis, The Netherlands)	N	NPS	80	80.00%	100%		
	Veritor SARS-CoV-2 (BectonDickinson, New Jersey, USA)	N	NPS	40	77.50%	NR		
	Panbio™ COVID-19 Ag RDT (Abbott, Jena, Germany)	N	NPS	40	70.00%	NR		
57	LIAISON SARS-CoV-2 Ag (DiaSorin, Saluggia, Italy)	N	NPS	414	67.70%	100%	Belgium	[75]
58	SARS-CoV-2 Rapid Ag Test (Roche, Basel, Switzerland)	N	NPS and TS	970	84.90%	99.50%	The Netherlands	[76]
59	Panbio™ COVID-19 Ag RDT (Abbott, Jena, Germany)	N	NPS	744	82.35%	100%	Greece	[77]
60	QuickNaviTM COVID19 Ag (Otsuka, Japan)	N	NPS	1934	80.30%	100%	Japan	[78]
61	Panbio™ COVID-19 Ag RDT (Abbott, Jena, Germany)	N	NPS	692	63.50%	100%	France	[79]
62	BinaxNOW™ COVID-19 Ag Self-Test (Abbott, Jena, Germany)	N	NPS	2308	66.70%	95.20%	United States	[80]
63	BinaxNOW™ COVID-19 Ag Self-Test (Abbott, Jena, Germany)	N	NPS	878	93.30%	99.90%	United States	[81]
64	SARS-CoV-2 Rapid Ag Test (Roche, Basel, Switzerland)	N	NPS	1465	65.30%	99.90%	Switzerland	[82]
65	Panbio™ COVID-19 Ag RDT (Abbott, Jena, Germany)	N	NPS and TS	105	NR	99·30%	Germany	[83]
	SARS-CoV-2 Rapid Ag Test (Roche, Basel, Switzerland)	N	NPS	115	NR	98.50%		
66	STANDARD F COVID-19 Ag FIA (SD Biosensor, Seoul, South Korea)	N	NPS	354	38.00%	99.00%	India	[84]
67	Elecsys^®^ SARS-CoV-2 Ag (Roche, Basel, Switzerland)	N	NPS and TS	3139	60.20%	99.90%	Germany	[85]
68	LIAISON SARS-CoV-2 Ag (DiaSorin, Saluggia, Italy)	N	NPS	378	84.80%	99.40%	France	[86]
69	Panbio™ COVID-19 Ag RDT (Abbott, Jena, Germany)	N	NPS	357	70.50%	100%	Spain	[87]
70	Biosynex COVID-19 Ag BSS Test (Biosynex, Strasbourg, France)	N	NPS	308	87.90%	98.50%	China	[88]
71	Lumipulse G SARS-CoV-2 Ag (Fujirebio, Tokyo, Japan)	N	NPS	410	91.70%	97.30%	Germany	[89]
	LIAISON SARS-CoV-2 Ag (DiaSorin, Saluggia, Italy)	N	NPS	408	99.10%	98.70%		
	Elecsys^®^ SARS-CoV-2 Ag (Roche, Basel, Switzerland)	N	NPS	410	65.50%	99.80%		
	SARS-CoV-2 Rapid Ag Test (Roche, Basel, Switzerland)	N	NPS	410	93.60%	100%		
72	SARS-CoV-2 Rapid Ag Test (Roche, Basel, Switzerland)	N	NPS and TS	2288	71.43%	99.68%	Spain	[90]
73	SARS-CoV-2 Ag Test (LumiraDx GmbH, Cologne, Germany)	N	NPS	761	82.20%	99.30%	Germany	[91]
74	Lumipulse G SARS-CoV-2 Ag (Fujirebio, Tokyo, Japan)	N	NPS	201	87.90%	95.80%	Italy	[92]
	STANDARD F COVID-19 Ag FIA (SD Biosensor, Seoul, South Korea)	N	NPS	93	35.70%	100%		
75	Sofia SARS Ag FIA (Quidel, San Diego, USA)	N	NPS	2887	76.60%	99.70%	United States	[93]
76	Elecsys^®^ SARS-CoV-2 Ag (Roche, Basel, Switzerland)	N	NPS	403	26.00%	100%	Italy	[94]
77	Panbio™ COVID-19 Ag RDT (Abbott, Jena, Germany)	N	NPS	1577	76.80%	100%	The Netherlands	[95]
78	Panbio™ COVID-19 Ag RDT (Abbott, Jena, Germany)	N	NPS	448	85.00%	100%	Spain	[96]
79	SARS-CoV-2 Ag Card (Biotical Health, Madrid, Spain)	N	NPS	188	66.70%	98.90%	Belgium	[97]
	Panbio™ COVID-19 Ag RDT (Abbott, Jena, Germany)	N	NPS	188	67.70%	100%		
	SARS-CoV-2 Rapid Ag Test (Roche, Basel, Switzerland)	N	NPS	188	69.80%	100%		
	VITROS SARS-CoV-2 Ag (Ortho, New Jersey, USA)	N	NPS	188	83.30%	100%		
80	COVID-VIRO (AAZ-LMB Boulogne-Billancourt, France)	N	NPS	234	96.88%	100%	France	[98]
81	Sofia SARS Ag FIA (Quidel, San Diego, USA)	N	NPS	64	93.80%	96.90%	Chile	[99]
	STANDARD F COVID-19 Ag FIA (SD Biosensor, Seoul, South Korea)	N	NPS	64	90.60%	96.90%		
82	NADAL COVID-19 Ag (Nal Von Minden GmbH, Moers, Germany)	N	NPS	124	73.10%	100%	Germany	[100]
83	STANDARD F COVID-19 Ag FIA (SD Biosensor, Seoul, South Korea)	N	NPS	2898	54.95%	97.80%	Italy	[101]
84	VITROS SARS-CoV-2 Ag (Ortho, New Jersey, USA)	N	NPS	24	84.20%	100%	China	[102]
85	STANDARD Q COVID-19 Ag Home Test (SD Biosensor, Seoul, South Korea)	N	NPS	146	82.50%	100%	Germany	[103]
86	Veritor SARS-CoV-2 (BectonDickinson, New Jersey, USA)	N	NPS	248	73.00%	100%	The Netherlands	[104]
87	BinaxNOW™ COVID-19 Ag Self-Test (Abbott, Jena, Germany)	N	NS	3302	100%	98.60%	Brazil	[105]
88	SARS-CoV-2 Ag Test (LumiraDx GmbH, Cologne, Germany)	N	NPS	792	95.20%	79.20%	Italy	[106]
89	STANDARD Q COVID-19 Ag Home Test (SD Biosensor, Seoul, South Korea)	N	NPS	532	41.20%	99.70%	Switzerland	[107]
	Panbio™ COVID-19 Ag RDT (Abbott, Jena, Germany)	N	NPS	532	41.20%	99.50%		
	Veritor SARS-CoV-2 (BectonDickinson, New Jersey, USA)	N	NPS	532	41.20%	99.70%		
90	Panbio™ COVID-19 Ag RDT (Abbott, Jena, Germany)	N	NPS	2413	NR	99.96%	Australia	[108]
91	STANDARD Q COVID-19 Ag Home Test (SD Biosensor, Seoul, South Korea)	N	NPS and TS	83	78.20%	64.20%	Egypt	[109]
92	Veritor SARS-CoV-2 (BectonDickinson, New Jersey, USA)	N	NPS and TS	2678	63.90%	99.60%	The Netherlands	[110]
	SARS-CoV-2 Rapid Ag Test (Roche, Basel, Switzerland)	N	NPS and TS	1370	62.90%	99.50%		
93	Panbio™ COVID-19 Ag RDT (Abbott, Jena, Germany)	N	NPS	958	90.50%	98.80%	Spain	[111]
94	BinaxNOW™ COVID-19 Ag Self-Test (Abbott, Jena, Germany)	N	NS	2110	77.2%	99.6%	United States	[112]

N = nucleocapsid protein; NPS = Nasopharyngeal swab; S = Saliva; NS = Nasal swab; TS = Throat swab; NR = Not reported; NPA = Nasopharyngeal aspirate; SP = Sputum; Ag = antigen; RDT = rapid diagnostic test.

**Table 3:** Summary of subgroups analysis of the sensitivity and specificity of rapid antigen tests for SARS-CoV-2.

Subgroups	Number of Studies	Total Number of Patients	Sensitivity [95% CI]	Specificity [95% CI]
**All studies**	94	74445	0.70 [0.69–0.71]	0.98 [0.98–0.98]
**Specimens**				
Nasopharyngeal swabs	58	38548	0.71 [0.70–0.72]	0.98 [0.98–0.99]
Nasal swabs	4	4258	0.83 [0.80–0.86]	0.98 [0.98–0.99]
Throat swabs	2	3623	0.69 [0.63–0.75]	0.99 [0.99–1.00]
Saliva	2	432	0.68 [0.59–0.77]	0.97 [0.95–0.99]
**Symptoms**				
Symptomatic	30	24726	0.82 [0.82–0.82]	0.98 [0.98–0.98]
Asymptomatic	14	14926	0.68 [0.65–0.71]	0.99 [0.99–0.99]
**Ct values**				
Ct value ≤25	13	5378	0.96 [0.95–0.97]	0.99 [0.98–0.99]
Ct value >25	12	6139	0.69 [0.67–0.71]	0.97 [0.96–0.97]
**Countries**				
Germany	14	14179	0.58 [0.56–0.60]	0.98 [0.98–0.98]
Spain	11	9391	0.71 [0.68–0.73]	0.99 [0.99–1.00]
Italy	10	11752	0.81 [0.79–0.83]	0.96 [0.96–0.97]
United States	8	10605	0.77 [0.75–0.79]	0.97 [0.97–0.97]
Japan	7	5192	0.72 [0.70–0.75]	0.99 [0.99–1.00]
Netherlands	7	8073	0.72 [0.70–0.75]	1.00 [0.99–1.00]
France	6	1858	0.58 [0.54–0.62]	1.00 [0.99–1.00]
Belgium	5	1634	0.73 [0.70–0.76]	0.84 [0.81–0.86]
Switzerland	3	2399	0.65 [0.61–0.70]	1.00 [1.00–1.00]
Chile	3	906	0.77 [0.69–0.85]	1.00 [0.99–1.00]
China	3	332	0.87 [0.77–0.97]	0.99 [0.97–1.00]
India	2	838	0.58 [0.52–0.63]	0.99 [0.98–1.00]

**Table 4:** Summary of subgroups analysis excluding case–control studies.

Subgroups	Number of Studies	Total Number of Patients	Sensitivity [95% CI]	Specificity [95% CI]
**All studies**	78	47415	0.72 [0.71–0.73]	0.98 [0.98–0.98]
**Specimens**				
Nasopharyngeal swabs	49	37646	0.71 [0.70–0.72]	0.98 [0.97–0.99]
Nasal swabs	3	3879	0.83 [0.80–0.85]	0.98 [0.98–0.99]
Throat swabs	2	3623	0.69 [0.63–0.75]	0.99 [0.99–1.00]
Saliva	2	432	0.68 [0.59–0.77]	0.97 [0.95–0.99]
**Symptoms**				
Symptomatic	24	21029	0.80 [0.78–0.81]	0.99 [0.99–0.99]
Asymptomatic	14	14926	0.68 [0.65–0.71]	0.99 [0.99–0.99]
**Ct values**				
Ct value ≤25	13	5378	0.96 [0.95–0.97]	0.99 [0.98–0.99]
Ct value >25	10	5693	0.69 [0.67–0.71]	0.97 [0.96–0.97]

**Table 5:** Comparative performance of studies that blinded the index test and studies that did not blind the index test.

Subgroups	Number of Studies	Total Number of Patients	Sensitivity [95% CI]	Specificity [95% CI]
**Studies (blinded the index test)**	30	24,470	0.72 [0.71–0.73]	0.97 [0.97–0.98]
**Studies (did not blind the index test)**	64	49,975	0.69 [0.68–0.70]	0.99 [0.99–0.99]

## Data Availability

Not applicable.

## Supplementary Materials

The following are available online at https://www.mdpi.com/article/10.3390/diagnostics12010110/s1, Table S1: QUADAS checklist.

## References

[1] Zhou P., Yang X.-L., Wang X.-G., Hu B., Zhang L., Zhang W., Si H.-R., Zhu Y., Li B., Huang C.-L. (2020). A Pneumonia Outbreak Associated with a New Coronavirus of Probable Bat Origin. Nature.

[2] Zhu N., Zhang D., Wang W., Li X., Yang B., Song J., Zhao X., Huang B., Shi W., Lu R. (2020). A Novel Coronavirus from Patients with Pneumonia in China, 2019. N. Engl. J. Med..

[3] Huang C., Wang Y., Li X., Ren L., Zhao J., Hu Y., Zhang L., Fan G., Xu J., Gu X. (2020). Clinical Features of Patients Infected with 2019 Novel Coronavirus in Wuhan, China. Lancet.

[4] Saniasiaya J., Islam M.A., Abdullah B. (2021). Prevalence of Olfactory Dysfunction in Coronavirus Disease 2019 (COVID-19): A Meta-analysis of 27,492 Patients. Laryngoscope.

[5] Saniasiaya J., Islam M.A., Abdullah B. (2021). Prevalence and Characteristics of Taste Disorders in Cases of COVID-19: A Meta-Analysis of 29,349 Patients. Otolaryngol.—Head Neck Surg..

[6] Subali A.D., Wiyono L. (2021). Reverse Transcriptase Loop Mediated Isothermal AmplifiCation (RT-LAMP) for COVID-19 Diagnosis: A Systematic Review and Meta-Analysis. Pathog. Glob. Health.

[7] Liu J., Liao X., Qian S., Yuan J., Wang F., Liu Y., Wang Z., Wang F.-S., Liu L., Zhang Z. (2020). Community Transmission of Severe Acute Respiratory Syndrome Coronavirus 2, Shenzhen, China, 2020. Emerg. Infect. Dis..

[8] Cheng V.C.C., Wong S.-C., Chen J.H.K., Yip C.C.Y., Chuang V.W.M., Tsang O.T.Y., Sridhar S., Chan J.F.W., Ho P.-L., Yuen K.-Y. (2020). Escalating Infection Control Response to the Rapidly Evolving Epidemiology of the Coronavirus Disease 2019 (COVID-19) Due to SARS-CoV-2 in Hong Kong. Infect. Control Hosp. Epidemiol..

[9] Asadi S., Bouvier N., Wexler A.S., Ristenpart W.D. (2020). The Coronavirus Pandemic and Aerosols: Does COVID-19 Transmit via Expiratory Particles?. Aerosol Sci. Technol..

[10] Pan K.-Y., Kok A.A.L., Eikelenboom M., Horsfall M., Jörg F., Luteijn R.A., Rhebergen D., van Oppen P., Giltay E.J., Penninx B.W.J.H. (2021). The Mental Health Impact of the COVID-19 Pandemic on People with and without Depressive, Anxiety, or Obsessive-Compulsive Disorders: A Longitudinal Study of Three Dutch Case-Control Cohorts. Lancet Psychiatry.

[11] Pierce M., Hope H., Ford T., Hatch S., Hotopf M., John A., Kontopantelis E., Webb R., Wessely S., McManus S. (2020). Mental Health before and during the COVID-19 Pandemic: A Longitudinal Probability Sample Survey of the UK Population. Lancet Psychiatry.

[12] Mofijur M., Fattah I.M.R., Alam M.A., Islam A.B.M.S., Ong H.C., Rahman S.M.A., Najafi G., Ahmed S.F., Uddin M.A., Mahlia T.M.I. (2021). Impact of COVID-19 on the Social, Economic, Environmental and Energy Domains: Lessons Learnt from a Global Pandemic. Sustain. Prod. Consum..

[13] Lu X., Wang L., Sakthivel S.K., Whitaker B., Murray J., Kamili S., Lynch B., Malapati L., Burke S.A., Harcourt J. (2020). US CDC Real-Time Reverse Transcription PCR Panel for Detection of Severe Acute Respiratory Syndrome Coronavirus 2. Emerg. Infect. Dis..

[14] Shen M., Zhou Y., Ye J., Abdullah AL-maskri A.A., Kang Y., Zeng S., Cai S. (2020). Recent Advances and Perspectives of Nucleic Acid Detection for Coronavirus. J. Pharm. Anal..

[15] Barbić L., Savić V., Bogdanić M., Antolašić L., Milašinčić L., Hruškar Ž., Sabadi D., Perić L., Tabain I., Stevanović V. (2020). Diagnosis of SARS-CoV-2 Infection. Infektološki Glas.

[16] Brümmer L.E., Katzenschlager S., Gaeddert M., Erdmann C., Schmitz S., Bota M., Grilli M., Larmann J., Weigand M.A., Pollock N.R. (2021). Accuracy of Novel Antigen Rapid Diagnostics for SARS-CoV-2: A Living Systematic Review and Meta-Analysis. PLoS Med..

[17] Khandker S.S., Nik Hashim N.H.H., Deris Z.Z., Shueb R.H., Islam M.A. (2021). Diagnostic Accuracy of Rapid Antigen Test Kits for Detecting SARS-CoV-2: A Systematic Review and Meta-Analysis of 17,171 Suspected COVID-19 Patients. J. Clin. Med..

[18] Liberati A., Altman D.G., Tetzlaff J., Mulrow C., Gotzsche P.C., Ioannidis J.P.A., Clarke M., Devereaux P.J., Kleijnen J., Moher D. (2009). The PRISMA Statement for Reporting Systematic Reviews and Meta-Analyses of Studies That Evaluate Healthcare Interventions: Explanation and Elaboration. BMJ.

[19] Whiting P.F., Rutjes A.W.S., Westwood M.E., Mallett S., Deeks J.J., Reitsma J.B., Leeflang M.M.G., Sterne J.A.C., Bossuyt P.M.M. (2011). QUADAS-2: A Revised Tool for the Quality Assessment of Diagnostic Accuracy Studies. Ann. Intern. Med..

[20] Chaimayo C., Kaewnaphan B., Tanlieng N., Athipanyasilp N., Sirijatuphat R., Chayakulkeeree M., Angkasekwinai N., Sutthent R., Puangpunngam N., Tharmviboonsri T. (2020). Rapid SARS-CoV-2 Antigen Detection Assay in Comparison with Real-Time RT-PCR Assay for Laboratory Diagnosis of COVID-19 in Thailand. Virol. J..

[21] Mak G.C., Cheng P.K., Lau S.S., Wong K.K., Lau C., Lam E.T., Chan R.C., Tsang D.N. (2020). Evaluation of Rapid Antigen Test for Detection of SARS-CoV-2 Virus. J. Clin. Virol..

[22] Scohy A., Anantharajah A., Bodéus M., Kabamba-Mukadi B., Verroken A., Rodriguez-Villalobos H. (2020). Low Performance of Rapid Antigen Detection Test as Frontline Testing for COVID-19 Diagnosis. J. Clin. Virol..

[23] Nagura-Ikeda M., Imai K., Tabata S., Miyoshi K., Murahara N., Mizuno T., Horiuchi M., Kato K., Imoto Y., Iwata M. (2020). Clinical Evaluation of Self-Collected Saliva by Quantitative Reverse Transcription-PCR (RT-QPCR), Direct RT-QPCR, Reverse Transcription—Loop-Mediated Isothermal Amplification, and a Rapid Antigen Test To Diagnose COVID-19. J. Clin. Microbiol..

[24] Porte L., Legarraga P., Vollrath V., Aguilera X., Munita J.M., Araos R., Pizarro G., Vial P., Iruretagoyena M., Dittrich S. (2020). Evaluation of a Novel Antigen-Based Rapid Detection Test for the Diagnosis of SARS-CoV-2 in Respiratory Samples. Int. J. Infect. Dis..

[25] Krüttgen A., Cornelissen C.G., Dreher M., Hornef M.W., Imöhl M., Kleines M. (2021). Comparison of the SARS-CoV-2 Rapid Antigen Test to the Real Star Sars-CoV-2 RT PCR Kit. J. Virol. Methods.

[26] Albert E., Torres I., Bueno F., Huntley D., Molla E., Fernández-Fuentes M.Á., Martínez M., Poujois S., Forqué L., Valdivia A. (2021). Field Evaluation of a Rapid Antigen Test (Panbio^TM^ COVID-19 Ag Rapid Test Device) for COVID-19 Diagnosis in Primary Healthcare Centres. Clin. Microbiol. Infect..

[27] Torres I., Poujois S., Albert E., Colomina J., Navarro D. (2021). Evaluation of a Rapid Antigen Test (Panbio^TM^ COVID-19 Ag Rapid Test Device) for SARS-CoV-2 Detection in Asymptomatic Close Contacts of COVID-19 Patients. Clin. Microbiol. Infect..

[28] Pray I.W., Ford L., Cole D., Lee C., Bigouette J.P., Abedi G.R., Bushman D., Delahoy M.J., Currie D., Cherney B. (2021). Performance of an Antigen-Based Test for Asymptomatic and Symptomatic SARS-CoV-2 Testing at Two University Campuses—Wisconsin, September–October 2020. MMWR. Morb. Mortal. Wkly. Rep..

[29] Diao B., Wen K., Zhang J., Chen J., Han C., Chen Y., Wang S., Deng G., Zhou H., Wu Y. (2021). Accuracy of a Nucleocapsid Protein Antigen Rapid Test in the Diagnosis of SARS-CoV-2 Infection. Clin. Microbiol. Infect..

[30] Lambert-Niclot S., Cuffel A., Le Pape S., Vauloup-Fellous C., Morand-Joubert L., Roque-Afonso A.-M., Le Goff J., Delaugerre C. (2020). Evaluation of a Rapid Diagnostic Assay for Detection of SARS-CoV-2 Antigen in Nasopharyngeal Swabs. J. Clin. Microbiol..

[31] Linares M., Pérez-Tanoira R., Carrero A., Romanyk J., Pérez-García F., Gómez-Herruz P., Arroyo T., Cuadros J. (2020). Panbio Antigen Rapid Test Is Reliable to Diagnose SARS-CoV-2 Infection in the First 7 Days after the Onset of Symptoms. J. Clin. Virol..

[32] Osterman A., Baldauf H.-M., Eletreby M., Wettengel J.M., Afridi S.Q., Fuchs T., Holzmann E., Maier A., Döring J., Grzimek-Koschewa N. (2021). Evaluation of Two Rapid Antigen Tests to Detect SARS-CoV-2 in a Hospital Setting. Med. Microbiol. Immunol..

[33] Aoki K., Nagasawa T., Ishii Y., Yagi S., Okuma S., Kashiwagi K., Maeda T., Miyazaki T., Yoshizawa S., Tateda K. (2021). Clinical Validation of Quantitative SARS-CoV-2 Antigen Assays to Estimate SARS-CoV-2 Viral Loads in Nasopharyngeal Swabs. J. Infect. Chemother..

[34] Nalumansi A., Lutalo T., Kayiwa J., Watera C., Balinandi S., Kiconco J., Nakaseegu J., Olara D., Odwilo E., Serwanga J. (2021). Field Evaluation of the Performance of a SARS-CoV-2 Antigen Rapid Diagnostic Test in Uganda Using Nasopharyngeal Samples. Int. J. Infect. Dis..

[35] Pollock N.R., Savage T.J., Wardell H., Lee R.A., Mathew A., Stengelin M., Sigal G.B. (2021). Correlation of SARS-CoV-2 Nucleocapsid Antigen and RNA Concentrations in Nasopharyngeal Samples from Children and Adults Using an Ultrasensitive and Quantitative Antigen Assay. J. Clin. Microbiol..

[36] Villaverde S., Domínguez-Rodríguez S., Sabrido G., Pérez-Jorge C., Plata M., Romero M.P., Grasa C.D., Jiménez A.B., Heras E., Broncano A. (2021). Diagnostic Accuracy of the Panbio Severe Acute Respiratory Syndrome Coronavirus 2 Antigen Rapid Test Compared with Reverse-Transcriptase Polymerase Chain Reaction Testing of Nasopharyngeal Samples in the Pediatric Population. J. Pediatr..

[37] Kobayashi R., Murai R., Asanuma K., Fujiya Y., Takahashi S. (2021). Evaluating a Novel, Highly Sensitive, and Quantitative Reagent for Detecting SARS-CoV-2 Antigen. J. Infect. Chemother..

[38] Blairon L., Cupaiolo R., Thomas I., Piteüs S., Wilmet A., Beukinga I., Tré-Hardy M. (2021). Efficacy Comparison of Three Rapid Antigen Tests for SARS-CoV-2 and How Viral Load Impact Their Performance. J. Med. Virol..

[39] Dankova Z., Novakova E., Skerenova M., Holubekova V., Lucansky V., Dvorska D., Brany D., Kolkova Z., Strnadel J., Mersakova S. (2021). Comparison of SARS-CoV-2 Detection by Rapid Antigen and by Three Commercial RT-QPCR Tests: A Study from Martin University Hospital in Slovakia. Int. J. Environ. Res. Public Health.

[40] Korenkov M., Poopalasingam N., Madler M., Vanshylla K., Eggeling R., Wirtz M., Fish I., Dewald F., Gieselmann L., Lehmann C. (2021). Evaluation of a Rapid Antigen Test To Detect SARS-CoV-2 Infection and Identify Potentially Infectious Individuals. J. Clin. Microbiol..

[41] Pérez-García F., Romanyk J., Gómez-Herruz P., Arroyo T., Pérez-Tanoira R., Linares M., Pérez Ranz I., Labrador Ballestero A., Moya Gutiérrez H., Ruiz-Álvarez M.J. (2021). Diagnostic Performance of CerTest and Panbio Antigen Rapid Diagnostic Tests to Diagnose SARS-CoV-2 Infection. J. Clin. Virol..

[42] Caputo V., Bax C., Colantoni L., Peconi C., Termine A., Fabrizio C., Calvino G., Luzzi L., Panunzi G.G., Fusco C. (2021). Comparative Analysis of Antigen and Molecular Tests for the Detection of Sars-CoV-2 and Related Variants: A Study on 4266 Samples. Int. J. Infect. Dis..

[43] Mayanskiy N., Brzhozovskaya E., Fedorova N., Lebedin Y. (2021). Parallel Detection of SARS-CoV-2 RNA and Nucleocapsid Antigen in Nasopharyngeal Specimens from a COVID-19 Patient Screening Cohort. Int. J. Infect. Dis..

[44] Gili A., Paggi R., Russo C., Cenci E., Pietrella D., Graziani A., Stracci F., Mencacci A. (2021). Evaluation of Lumipulse® G SARS-CoV-2 Antigen Assay Automated Test for Detecting SARS-CoV-2 Nucleocapsid Protein (NP) in Nasopharyngeal Swabs for Community and Population Screening. Int. J. Infect. Dis..

[45] Pickering S., Batra R., Merrick B., Snell L.B., Nebbia G., Douthwaite S., Reid F., Patel A., Kia Ik M.T., Patel B. (2021). Comparative Performance of SARS-CoV-2 Lateral Flow Antigen Tests and Association with Detection of Infectious Virus in Clinical Specimens: A Single-Centre Laboratory Evaluation Study. The Lancet Microbe.

[46] Hirotsu Y., Sugiura H., Maejima M., Hayakawa M., Mochizuki H., Tsutsui T., Kakizaki Y., Miyashita Y., Omata M. (2021). Comparison of Roche and Lumipulse Quantitative SARS-CoV-2 Antigen Test Performance Using Automated Systems for the Diagnosis of COVID-19. Int. J. Infect. Dis..

[47] Asai N., Sakanashi D., Ohashi W., Nakamura A., Kawamoto Y., Miyazaki N., Ohno T., Yamada A., Chida S., Shibata Y. (2021). Efficacy and Validity of Automated Quantitative Chemiluminescent Enzyme Immunoassay for SARS-CoV-2 Antigen Test from Saliva Specimen in the Diagnosis of COVID-19. J. Infect. Chemother..

[48] Ngo Nsoga M.T., Kronig I., Perez Rodriguez F.J., Sattonnet-Roche P., Da Silva D., Helbling J., Sacks J.A., de Vos M., Boehm E., Gayet- Ageron A. (2021). Diagnostic Accuracy of Panbio Rapid Antigen Tests on Oropharyngeal Swabs for Detection of SARS-CoV-2. PLoS ONE.

[49] Häuser F., Sprinzl M.F., Dreis K.J., Renzaho A., Youhanen S., Kremer W.M., Podlech J., Galle P.R., Lackner K.J., Rossmann H. (2021). Evaluation of a Laboratory-Based High-Throughput SARS-CoV-2 Antigen Assay for Non-COVID-19 Patient Screening at Hospital Admission. Med. Microbiol. Immunol..

[50] Van Honacker E., Van Vaerenbergh K., Boel A., De Beenhouwer H., Leroux-Roels I., Cattoir L. (2021). Comparison of Five SARS-CoV-2 Rapid Antigen Detection Tests in a Hospital Setting and Performance of One Antigen Assay in Routine Practice: A Useful Tool to Guide Isolation Precautions?. J. Hosp. Infect..

[51] Takeuchi Y., Akashi Y., Kato D., Kuwahara M., Muramatsu S., Ueda A., Notake S., Nakamura K., Ishikawa H., Suzuki H. (2021). Diagnostic Performance and Characteristics of Anterior Nasal Collection for the SARS-CoV-2 Antigen Test: A Prospective Study. Sci. Rep..

[52] Terpos E., Ntanasis-Stathopoulos I., Skvarč M. (2021). Clinical Application of a New SARS-CoV-2 Antigen Detection Kit (Colloidal Gold) in the Detection of COVID-19. Diagnostics.

[53] Fiedler M., Holtkamp C., Dittmer U., Anastasiou O.E. (2021). Performance of the LIAISON® SARS-CoV-2 Antigen Assay vs. SARS-CoV-2-RT-PCR. Pathogens.

[54] Leixner G., Voill-Glaninger A., Bonner E., Kreil A., Zadnikar R., Viveiros A. (2021). Evaluation of the AMP SARS-CoV-2 Rapid Antigen Test in a Hospital Setting. Int. J. Infect. Dis..

[55] Krüger L.J., Gaeddert M., Tobian F., Lainati F., Gottschalk C., Klein J.A.F., Schnitzler P., Kräusslich H.-G., Nikolai O., Lindner A.K. (2021). The Abbott PanBio WHO Emergency Use Listed, Rapid, Antigen-Detecting Point-of-Care Diagnostic Test for SARS-CoV-2—Evaluation of the Accuracy and Ease-of-Use. PLoS ONE.

[56] Mboumba Bouassa R.-S., Veyer D., Péré H., Bélec L. (2021). Analytical Performances of the Point-of-Care SIENNA^TM^ COVID-19 Antigen Rapid Test for the Detection of SARS-CoV-2 Nucleocapsid Protein in Nasopharyngeal Swabs: A Prospective Evaluation during the COVID-19 Second Wave in France. Int. J. Infect. Dis..

[57] Ciotti M., Maurici M., Pieri M., Andreoni M., Bernardini S. (2021). Performance of a Rapid Antigen Test in the Diagnosis of SARS-CoV-2 Infection. J. Med. Virol..

[58] Kolwijck E., Brouwers-Boers M., Broertjes J., van Heeswijk K., Runderkamp N., Meijer A., Hermans M.H.A., Leenders A.C.A.P. (2021). Validation and Implementation of the Panbio COVID-19 Ag Rapid Test for the Diagnosis of SARS-CoV-2 Infection in Symptomatic Hospital Healthcare Workers. Infect. Prev. Pract..

[59] Salvagno G.L., Gianfilippi G., Bragantini D., Henry B.M., Lippi G. (2021). Clinical Assessment of the Roche SARS-CoV-2 Rapid Antigen Test. Diagnosis.

[60] Bianco G., Boattini M., Barbui A.M., Scozzari G., Riccardini F., Coggiola M., Lupia E., Cavallo R., Costa C. (2021). Evaluation of an Antigen-Based Test for Hospital Point-of-Care Diagnosis of SARS-CoV-2 Infection. J. Clin. Virol..

[61] Peña M., Ampuero M., Garcés C., Gaggero A., García P., Velasquez M.S., Luza R., Alvarez P., Paredes F., Acevedo J. (2021). Performance of SARS-CoV-2 Rapid Antigen Test Compared with Real-Time RT-PCR in Asymptomatic Individuals. Int. J. Infect. Dis..

[62] Peña-Rodríguez M., Viera-Segura O., García-Chagollán M., Zepeda-Nuño J.S., Muñoz-Valle J.F., Mora-Mora J., Espinoza-De León G., Bustillo-Armendáriz G., García-Cedillo F., Vega-Magaña N. (2021). Performance Evaluation of a Lateral Flow Assay for Nasopharyngeal Antigen Detection for SARS-CoV-2 Diagnosis. J. Clin. Lab. Anal..

[63] Ristić M., Nikolić N., Čabarkapa V., Turkulov V., Petrović V. (2021). Validation of the STANDARD Q COVID-19 antigen test in Vojvodina, Serbia. PLoS ONE.

[64] Pérez-García F., Romanyk J., Moya Gutiérrez H., Labrador Ballestero A., Pérez Ranz I., González Arroyo J., González Ventosa V., Pérez-Tanoira R., Domingo Cruz C., Cuadros-González J. (2021). Comparative Evaluation of Panbio and SD Biosensor Antigen Rapid Diagnostic Tests for COVID-19 Diagnosis. J. Med. Virol..

[65] Kanaujia R., Ghosh A., Mohindra R., Singla V., Goyal K., Gudisa R., Sharma V., Mohan L., Kaur N., Mohi G.K. (2021). Rapid Antigen Detection Kit for the Diagnosis of SARS-CoV-2—Are We Missing Asymptomatic Patients?. Indian J. Med. Microbiol..

[66] Courtellemont L., Guinard J., Guillaume C., Giaché S., Rzepecki V., Seve A., Gubavu C., Baud K., Le Helloco C., Cassuto G.N. (2021). High Performance of a Novel Antigen Detection Test on Nasopharyngeal Specimens for Diagnosing SARS-CoV-2 Infection. J. Med. Virol..

[67] Bulilete O., Lorente P., Leiva A., Carandell E., Oliver A., Rojo E., Pericas P., Llobera J. (2021). Panbio^TM^ Rapid Antigen Test for SARS-CoV-2 Has Acceptable Accuracy in Symptomatic Patients in Primary Health Care. J. Infect..

[68] Van der Moeren N., Zwart V.F., Lodder E.B., Van den Bijllaardt W., Van Esch H.R.J.M., Stohr J.J.J.M., Pot J., Welschen I., Van Mechelen P.M.F., Pas S.D. (2021). Evaluation of the Test Accuracy of a SARS-CoV-2 Rapid Antigen Test in Symptomatic Community Dwelling Individuals in the Netherlands. PLoS ONE.

[69] Amendola A., Sberna G., Lalle E., Colavita F., Castilletti C., Menchinelli G., Posteraro B., Sanguinetti M., Ippolito G., Bordi L. (2021). Saliva Is a Valid Alternative to Nasopharyngeal Swab in Chemiluminescence-Based Assay for Detection of SARS-CoV-2 Antigen. J. Clin. Med..

[70] Berger A., Nsoga M.T.N., Perez-Rodriguez F.J., Aad Y.A., Sattonnet-Roche P., Gayet-Ageron A., Jaksic C., Torriani G., Boehm E., Kronig I. (2021). Diagnostic Accuracy of Two Commercial SARS-CoV-2 Antigen-Detecting Rapid Tests at the Point of Care in Community-Based Testing Centers. PLoS ONE.

[71] Osmanodja B., Budde K., Zickler D., Naik M.G., Hofmann J., Gertler M., Hülso C., Rössig H., Horn P., Seybold J. (2021). Accuracy of a Novel SARS-CoV-2 Antigen-Detecting Rapid Diagnostic Test from Standardized Self-Collected Anterior Nasal Swabs. J. Clin. Med..

[72] Wagenhäuser I., Knies K., Rauschenberger V., Eisenmann M., McDonogh M., Petri N., Andres O., Flemming S., Gawlik M., Papsdorf M. (2021). Clinical Performance Evaluation of SARS-CoV-2 Rapid Antigen Testing in Point of Care Usage in Comparison to RT-QPCR. EBioMedicine.

[73] Jääskeläinen A.E., Ahava M.J., Jokela P., Szirovicza L., Pohjala S., Vapalahti O., Lappalainen M., Hepojoki J., Kurkela S. (2021). Evaluation of Three Rapid Lateral Flow Antigen Detection Tests for the Diagnosis of SARS-CoV-2 Infection. J. Clin. Virol..

[74] Koeleman J.G.M., Brand H., de Man S.J., Ong D.S.Y. (2021). Clinical Evaluation of Rapid Point-of-Care Antigen Tests for Diagnosis of SARS-CoV-2 Infection. Eur. J. Clin. Microbiol. Infect. Dis..

[75] Lefever S., Indevuyst C., Cuypers L., Dewaele K., Yin N., Cotton F., Padalko E., Oyaert M., Descy J., Cavalier E. (2021). Comparison of the Quantitative DiaSorin Liaison Antigen Test to Reverse Transcription-PCR for the Diagnosis of COVID-19 in Symptomatic and Asymptomatic Outpatients. J. Clin. Microbiol..

[76] Iglὁi Z., Velzing J., van Beek J., van de Vijver D., Aron G., Ensing R., Benschop K., Han W., Boelsums T., Koopmans M. (2021). Clinical Evaluation of Roche SD Biosensor Rapid Antigen Test for SARS-CoV-2 in Municipal Health Service Testing Site, the Netherlands. Emerg. Infect. Dis..

[77] Eleftheriou I., Dasoula F., Dimopoulou D., Lebessi E., Serafi E., Spyridis N., Tsolia M. (2021). Real-life Evaluation of a COVID-19 Rapid Antigen Detection Test in Hospitalized Children. J. Med. Virol..

[78] Kiyasu Y., Takeuchi Y., Akashi Y., Kato D., Kuwahara M., Muramatsu S., Notake S., Ueda A., Nakamura K., Ishikawa H. (2021). Prospective Analytical Performance Evaluation of the QuickNavi^TM^-COVID19 Ag for Asymptomatic Individuals. J. Infect. Chemother..

[79] Ferté T., Ramel V., Cazanave C., Lafon M.-E., Bébéar C., Malvy D., Georges-Walryck A., Dehail P. (2021). Accuracy of COVID-19 Rapid Antigenic Tests Compared to RT-PCR in a Student Population: The StudyCov Study. J. Clin. Virol..

[80] Pollock N.R., Jacobs J.R., Tran K., Cranston A.E., Smith S., O’Kane C.Y., Roady T.J., Moran A., Scarry A., Carroll M. (2021). Performance and Implementation Evaluation of the Abbott BinaxNOW Rapid Antigen Test in a High-Throughput Drive-Through Community Testing Site in Massachusetts. J. Clin. Microbiol..

[81] Pilarowski G., Lebel P., Sunshine S., Liu J., Crawford E., Marquez C., Rubio L., Chamie G., Martinez J., Peng J. (2021). Performance Characteristics of a Rapid Severe Acute Respiratory Syndrome Coronavirus 2 Antigen Detection Assay at a Public Plaza Testing Site in San Francisco. J. Infect. Dis..

[82] Jegerlehner S., Suter-Riniker F., Jent P., Bittel P., Nagler M. (2021). Diagnostic Accuracy of a SARS-CoV-2 Rapid Antigen Test in Real-Life Clinical Settings. Int. J. Infect. Dis..

[83] Corman V.M., Haage V.C., Bleicker T., Schmidt M.L., Mühlemann B., Zuchowski M., Jo W.K., Tscheak P., Möncke-Buchner E., Müller M.A. (2021). Comparison of Seven Commercial SARS-CoV-2 Rapid Point-of-Care Antigen Tests: A Single-Centre Laboratory Evaluation Study. Lancet Microbe.

[84] Kiro V., Gupta A., Singh P., Sharad N., Khurana S., Parakash S., Dar L., Malhotra R., Wig N., Kumar A. (2021). Evaluation of COVID-19 Antigen Fluorescence Immunoassay Test for Rapid Detection of SARS-CoV-2. J. Glob. Infect. Dis..

[85] Nörz D., Olearo F., Perisic S., Bauer M.F., Riester E., Schneider T., Schönfeld K., Laengin T., Lütgehetmann M. (2021). Multicenter Evaluation of a Fully Automated High-Throughput SARS-CoV-2 Antigen Immunoassay. Infect. Dis. Ther..

[86] Hartard C., Berger S., Josse T., Schvoerer E., Jeulin H. (2021). Performance Evaluation of an Automated SARS-CoV-2 Ag Test for the Diagnosis of COVID-19 Infection on Nasopharyngeal Swabs. Clin. Chem. Lab. Med..

[87] Carbonell-Sahuquillo S., Lázaro-Carreño M.I., Camacho J., Barrés-Fernández A., Albert E., Torres I., Bretón-Martínez J.R., Martínez-Costa C., Navarro D. (2021). Evaluation of a Rapid Antigen Detection Test (Panbio^TM^ COVID-19 Ag Rapid Test Device) as a Point-of-care Diagnostic Tool for COVID-19 in a Pediatric Emergency Department. J. Med. Virol..

[88] Jung C., Levy C., Varon E., Biscardi S., Batard C., Wollner A., Deberdt P., Sellam A., Hau I., Cohen R. (2021). Diagnostic Accuracy of SARS-CoV-2 Antigen Detection Test in Children: A Real-Life Study. Front. Pediatr..

[89] Osterman A., Iglhaut M., Lehner A., Späth P., Stern M., Autenrieth H., Muenchhoff M., Graf A., Krebs S., Blum H. (2021). Comparison of Four Commercial, Automated Antigen Tests to Detect SARS-CoV-2 Variants of Concern. Med. Microbiol. Immunol..

[90] Fernandez-Montero A., Argemi J., Rodríguez J.A., Ariño A.H., Moreno-Galarraga L. (2021). Validation of a Rapid Antigen Test as a Screening Tool for SARS-CoV-2 Infection in Asymptomatic Populations. Sensitivity, Specificity and Predictive Values. EClinicalMedicine.

[91] Krüger L.J., Klein J.A.F., Tobian F., Gaeddert M., Lainati F., Klemm S., Schnitzler P., Bartenschlager R., Cerikan B., Neufeldt C.J. (2021). Evaluation of Accuracy, Exclusivity, Limit-of-Detection and Ease-of-Use of LumiraDx^TM^: An Antigen-Detecting Point-of-Care Device for SARS-CoV-2. medRxiv.

[92] Baccani I., Morecchiato F., Chilleri C., Cervini C., Gori E., Matarrese D., Bassetti A., Bonizzoli M., Mencarini J., Antonelli A. (2021). Evaluation of Three Immunoassays for the Rapid Detection of SARS-CoV-2 Antigens. Diagn. Microbiol. Infect. Dis..

[93] Smith R.D., Johnson J.K., Clay C., Girio-Herrera L., Stevens D., Abraham M., Zimand P., Ahlman M., Gimigliano S., Zhao R. (2021). Clinical Evaluation of Sofia Rapid Antigen Assay for Detection of Severe Acute Respiratory Syndrome Coronavirus 2 (SARS-CoV-2) among Emergency Department to Hospital Admissions. Infect. Control Hosp. Epidemiol..

[94] Mueller T., Kompatscher J., La Guardia M. (2021). Diagnostic Performance of the Elecsys SARS-CoV-2 Antigen Assay in the Clinical Routine of a Tertiary Care Hospital: Preliminary Results from a Single-center Evaluation. J. Clin. Lab. Anal..

[95] Gremmels H., Winkel B.M.F., Schuurman R., Rosingh A., Rigter N.A.M., Rodriguez O., Ubijaan J., Wensing A.M.J., Bonten M.J.M., Hofstra L.M. (2021). Real-Life Validation of the Panbio^TM^ COVID-19 Antigen Rapid Test (Abbott) in Community-Dwelling Subjects with Symptoms of Potential SARS-CoV-2 Infection. EClinicalMedicine.

[96] Escrivá B.F., Mochón M.D.O., González R.M., García C.S., Pla A.T., Ricart A.S., García M.M., Aranda I.T., García F.G., Cardona C.G. (2021). “The Effectiveness of Rapid Antigen Test-Based for SARS-CoV-2 Detection in Nursing Homes in Valencia, Spain”. J. Clin. Virol..

[97] Favresse J., Gillot C., Oliveira M., Cadrobbi J., Elsen M., Eucher C., Laffineur K., Rosseels C., Van Eeckhoudt S., Nicolas J.-B. (2021). Head-to-Head Comparison of Rapid and Automated Antigen Detection Tests for the Diagnosis of SARS-CoV-2 Infection. J. Clin. Med..

[98] Cassuto N.G., Gravier A., Colin M., Theillay A., Pires-Roteira D., Pallay S., Serreau R., Hocqueloux L., Prazuck T. (2021). Evaluation of a SARS-CoV-2 Antigen-detecting Rapid Diagnostic Test as a Self-test: Diagnostic Performance and Usability. J. Med. Virol..

[99] Porte L., Legarraga P., Iruretagoyena M., Vollrath V., Pizarro G., Munita J., Araos R., Weitzel T. (2021). Evaluation of Two Fluorescence Immunoassays for the Rapid Detection of SARS-CoV-2 Antigen—New Tool to Detect Infective COVID-19 Patients. PeerJ.

[100] Strömer A., Rose R., Schäfer M., Schön F., Vollersen A., Lorentz T., Fickenscher H., Krumbholz A. (2020). Performance of a Point-of-Care Test for the Rapid Detection of SARS-CoV-2 Antigen. Microorganisms.

[101] Menchinelli G., De Angelis G., Cacaci M., Liotti F.M., Candelli M., Palucci I., Santangelo R., Sanguinetti M., Vetrugno G., Franceschi F. (2021). SARS-CoV-2 Antigen Detection to Expand Testing Capacity for COVID-19: Results from a Hospital Emergency Department Testing Site. Diagnostics.

[102] Levett P.N., Cheung B., Kustra J., Pidduck T., Mak A., Tsang F., Petric M., Krajden M. (2021). Evaluation of a High Volume Antigen Test for Detection of SARS-CoV-2. J. Clin. Virol..

[103] Lindner A.K., Nikolai O., Rohardt C., Kausch F., Wintel M., Gertler M., Burock S., Hörig M., Bernhard J., Tobian F. (2021). Diagnostic Accuracy and Feasibility of Patient Self-Testing with a SARS-CoV-2 Antigen-Detecting Rapid Test. J. Clin. Virol..

[104] Van der Moeren N., Zwart V.F., Goderski G., Rijkers G.T., van den Bijllaardt W., Veenemans J., Kluytmans J.A.J.W., Pas S.D., Meijer A., Verweij J.J. (2021). Performance of the Diasorin SARS-CoV-2 Antigen Detection Assay on the LIAISON XL. J. Clin. Virol..

[105] Pilarowski G., Marquez C., Rubio L., Peng J., Martinez J., Black D., Chamie G., Jones D., Jacobo J., Tulier-Laiwa V. (2020). Field Performance and Public Health Response Using the BinaxNOW^TM^ Rapid Severe Acute Respiratory Syndrome Coronavirus 2 (SARS-CoV-2) Antigen Detection Assay During Community-Based Testing. Clin. Infect. Dis..

[106] Leli C., Di Matteo L., Gotta F., Cornaglia E., Vay D., Megna I., Pensato R.E., Boverio R., Rocchetti A. (2021). Performance of a SARS-CoV-2 Antigen Rapid Immunoassay in Patients Admitted to the Emergency Department. Int. J. Infect. Dis..

[107] Caruana G., Croxatto A., Kampouri E., Kritikos A., Opota O., Foerster M., Brouillet R., Senn L., Lienhard R., Egli A. (2021). Implementing SARS-CoV-2 Rapid Antigen Testing in the Emergency Ward of a Swiss University Hospital: The INCREASE Study. Microorganisms.

[108] Muhi S., Tayler N., Hoang T., Ballard S.A., Graham M., Rojek A., Kwong J.C., Trubiano J.A., Smibert O., Drewett G. (2021). Multi-Site Assessment of Rapid, Point-of-Care Antigen Testing for the Diagnosis of SARS-CoV-2 Infection in a Low-Prevalence Setting: A Validation and Implementation Study. Lancet Reg. Health-West. Pac..

[109] Amer R.M., Samir M., Gaber O.A., EL-Deeb N.A., Abdelmoaty A.A., Ahmed A.A., Samy W., Atta A.H., Walaa M., Anis R.H. (2021). Diagnostic Performance of Rapid Antigen Test for COVID-19 and the Effect of Viral Load, Sampling Time, Subject’s Clinical and Laboratory Parameters on Test Accuracy. J. Infect. Public Health.

[110] Schuit E., Veldhuijzen I.K., Venekamp R.P., van den Bijllaardt W., Pas S.D., Lodder E.B., Molenkamp R., GeurtsvanKessel C.H., Velzing J., Huisman R.C. (2021). Diagnostic Accuracy of Rapid Antigen Tests in Asymptomatic and Presymptomatic Close Contacts of Individuals with Confirmed SARS-CoV-2 Infection: Cross Sectional Study. BMJ.

[111] Merino P., Guinea J., Muñoz-Gallego I., González-Donapetry P., Galán J.C., Antona N., Cilla G., Hernáez-Crespo S., Díaz-de Tuesta J.L., Gual-de Torrella A. (2021). Multicenter Evaluation of the PanbioTM COVID-19 Rapid Antigen-Detection Test for the Diagnosis of SARS-CoV-2 Infection. Clin. Microbiol. Infect..

[112] Shah M.M., Salvatore P.P., Ford L., Kamitani E., Whaley M.J., Mitchell K., Currie D.W., Morgan C.N., Segaloff H.E., Lecher S. (2021). Performance of Repeat BinaxNOW Severe Acute Respiratory Syndrome Coronavirus 2 Antigen Testing in a Community Setting, Wisconsin, November 2020–December 2020. Clin. Infect. Dis..

[113] Mak G.C.K., Lau S.S.Y., Wong K.K.Y., Chow N.L.S., Lau C.S., Lam E.T.K., Chan R.C.W., Tsang D.N.C. (2021). Evaluation of Rapid Antigen Detection Kit from the WHO Emergency Use List for Detecting SARS-CoV-2. J. Clin. Virol..

[114] Kim D., Lee J.-Y., Yang J.-S., Kim J.W., Kim V.N., Chang H. (2020). The Architecture of SARS-CoV-2 Transcriptome. Cell.

[115] Surjit M., Lal S.K. (2008). The SARS-CoV Nucleocapsid Protein: A Protein with Multifarious Activities. Infect. Genet. Evol..

[116] Tsang N.N.Y., So H.C., Ng K.Y., Cowling B.J., Leung G.M., Ip D.K.M. (2021). Diagnostic Performance of Different Sampling Approaches for SARS-CoV-2 RT-PCR Testing: A Systematic Review and Meta-Analysis. Lancet Infect. Dis..

[117] Zhao L., Li L., Liu G., Chen L., Liu X., Zhu J., Li B. (2013). Effect of Freeze–Thaw Cycles on the Molecular Weight and Size Distribution of Gluten. Food Res. Int..

[118] Shah V.P., Farah W.H., Hill J.C., Hassett L.C., Binnicker M.J., Yao J.D., Murad M.H. (2021). Association Between SARS-CoV-2 Cycle Threshold Values and Clinical Outcomes in Patients With COVID-19: A Systematic Review and Meta-Analysis. Open Forum Infect. Dis..

